# Impact of Stress and Anxiety on Cardiovascular Health in Pregnancy: A Scoping Review

**DOI:** 10.3390/jcm14030909

**Published:** 2025-01-30

**Authors:** Brenda-Cristiana Bernad, Mirela-Cleopatra Tomescu, Dana Emilia Velimirovici, Minodora Andor, Diana Lungeanu, Virgil Enătescu, Adina-Ioana Bucur, Ana Lascu, Andreea-Luciana Raţă, Elena Silvia Bernad, Vlad Nicoraș, Diana-Aurora Arnăutu, Oana Neda-Stepan, Lavinia Hogea

**Affiliations:** 1 Doctoral School, “Victor Babes” University of Medicine and Pharmacy, 300041 Timisoara, Romania; bernad.brenda@umft.ro; 2 Center for Neuropsychology and Behavioral Medicine, “Victor Babes” University of Medicine and Pharmacy, 300041 Timisoara, Romania; hogea.lavinia@umft.ro; 3 Multidisciplinary Heart Research Center, “Victor Babes” University of Medicine and Pharmacy, 300041 Timisoara, Romania; tomescu.mirela@umft.ro (M.-C.T.); andor.minodora@umft.ro (M.A.); aurora.bordejevic@umft.ro (D.-A.A.); 4 Department of Internal Medicine, “Victor Babes” University of Medicine and Pharmacy, 300041 Timisoara, Romania; 5 Timisoara Municipal Clinical Emergency Hospital, 300040 Timisoara, Romania; 6 Department of Cardiology, “Victor Babeș” University of Medicine and Pharmacy, 300041 Timisoara, Romania; dana.velimirovici@umft.ro (D.E.V.); enatescu.virgil@umft.ro (V.E.); oana.neda-stepan@umft.ro (O.N.-S.); 7 Institute of Cardiovascular Diseases Timisoara, 300310 Timisoara, Romania; bucur.adina@umft.ro (A.-I.B.); lascu.ana@umft.ro (A.L.); 8 Center for Modeling Biological Systems and Data Analysis, “Victor Babes” University of Medicine and Pharmacy, 300041 Timisoara, Romania; dlungeanu@umft.ro; 9 Department of Functional Sciences, Faculty of Medicine, “Victor Babes” University of Medicine and Pharmacy, 300041 Timisoara, Romania; 10 Clinic of Psychiatry, “Pius Brinzeu” County Clinical Emergency Hospital, 300723 Timisoara, Romania; vnicoras@gmail.com; 11 Centre for Translational Research and Systems Medicine, “Victor Babes” University of Medicine and Pharmacy, 2 EftimieMurgu Square, 300041 Timisoara, Romania; 12 Department of Surgical Emergencies, “Victor Babes” University of Medicine and Pharmacy, 300041 Timisoara, Romania; andreea.rata@umft.ro; 13 Department of Vascular Surgery, “Pius Brinzeu” County Clinical Emergency Hospital, 300723 Timisoara, Romania; 14 Department of Obstetrics and Gynecology, “Victor Babes” University of Medicine and Pharmacy, 300041 Timisoara, Romania; 15 Ist Clinic of Obstetrics and Gynecology, “Pius Brinzeu” County Clinical Emergency Hospital, 300723 Timisoara, Romania; 16 Center for Laparoscopy, Laparoscopic Surgery and In Vitro Fertilization, “Victor Babes” University of Medicine and Pharmacy, 300041 Timisoara, Romania; 17 Department of Neuroscience, “Victor Babes” University of Medicine and Pharmacy, 300041 Timisoara, Romania

**Keywords:** pregnancy, foetus, stress, anxiety, cardiovascular diseases, heart disease risk factors, psychological, mental health

## Abstract

Complex biological processes that enable optimal foetal growth throughout pregnancy are linked to notable haemodynamic and metabolic changes in the mother’s body. An inability to adapt to these changes can affect cardiovascular health. During pregnancy, women may experience mood swings, anxiety, and emotional ambivalence. These symptoms can lead to stress and harm the mental well-being of expectant mothers. It is crucial to know the aspects that can influence the development of cardiovascular problems among pregnant women. Effective management requires identifying risk factors. Applying the PRISMA ScR guidelines, we conducted a scoping review to explore and summarise the evidence regarding the impact of stress and anxiety on cardiovascular health in pregnant women. The following enquiries were looked into as research topics: What effects do anxiety and stress have on a pregnant woman’s cardiovascular health? How is it quantifiable? It is essential to comprehend the physiological changes that the body undergoes throughout pregnancy in order to inform and assist both patients and medical professionals. This makes it possible for them to identify any pathological disorders or risk factors that could worsen the health of expectant mothers. Psychological and cardiovascular risk factor screening, either before or during pregnancy, may be able to uncover circumstances that require specific medical and psychological therapies in order to lower maternal morbidity and death from cardiovascular disease. Our findings underscore the need for systematic psychological and cardiovascular screening during prenatal care to mitigate adverse outcomes and improve maternal–foetal health.

## 1. Introduction

Hormonal changes in women of reproductive age may be related to specific cardiovascular manifestations, with women of childbearing age having a higher prevalence of cardiovascular disease (CVD) [1], which requires correct evaluation of the pregnant woman starting from the first visit and the monitoring of cardiovascular parameters throughout the pregnancy. An additional reason for careful monitoring is the fact that CVD is the leading cause of death for women worldwide [2]. Still, only 44% of American women identify heart health as their top health concern, according to a survey, despite numerous national programmes to increase public awareness of the issue [3,4].

The manifestations of CVD may occur the first time in pregnancy for, probably due to the hormonal and adaptive changes in the body during the pregnancy period, or they may occur prior to pregnancy [5]. Given that pregnancy is known to increase a woman’s risk of developing cardiovascular disease (CVD), it is crucial to properly assess and monitor pregnant women in order to lower the risk of complications during and after pregnancy. However, even experienced medical professionals may find it challenging to differentiate between symptoms of CVD and a normal pregnancy. Owing to these current inadequacies in healthcare, misdiagnosis by healthcare providers causes treatment delays. Most often, maternal death is related to hypertensive disease and its complications, cardiomyopathy, and other fatalities due to maternal CVD [6,7,8].

In the medical literature, Selye was the first to identify stress as the body’s non-specific (neuroendocrine) reaction to any unpleasant stimulus [9]. Later, this idea was clarified by separating the terms “stressor” and “stress response”, and stress was defined as the body’s reaction to environmental pressures that are more than it can naturally regulate [10]. When managed well, stress is seen as a necessary component of an individual’s motivation, adaptation, and response to the environment, all of which can have a biopsychosocial effect on their health [11]. However, excessive stress can affect the human body in a variety of ways, from changes in homeostasis to potentially fatal consequences [12]. A person’s internal perceptions can also add to stress, which, over time, can result in anxiety and other destructive emotions and experiences like pain and despair. It can even lead to significant psychological problems [13].

In mediating cardiovascular changes induced by stress, neuroendocrine and inflammatory pathways are described. The sympathetic nervous system and the hypothalamic–pituitary–adrenal axis are activated by stressful events, which cause a variety of neurochemical, neurotransmitter, and hormonal changes. Proinflammatory cytokine signalling, peripheral monocyte infiltration, microglial activation, and hypothalamic–pituitary–adrenal axis hyperactivity contribute to vulnerability to stress, according to research that demonstrates a clear link between the neuroendocrine and immune systems [14]. Endothelial dysfunction is caused by mental stress through activation of the sympathetic nervous system, the release of corticotropin-releasing hormone from the hypothalamus, the inhibition of nitric oxide synthesis by cortisol, and increased levels of proinflammatory cytokines [15].

In addition, emotional events can cause instability of cardiac repolarization due to an imbalance cardiac autonomic nerve stimulation, which can cause asymmetric repolarization and arrhythmia. Acute emotional stress can also cause elevated catecholamine release, leading to direct myocyte injury from calcium overload known as myocytolysis, coronary microvascular vasoconstriction, and increased left ventricular afterload [16].

Stress can disrupt normal physiological immune functions by compromising the control mechanism of self-tolerance, a situation in which the immune system turns into a self-antigen. Exposure to traumatic stress and post-traumatic stress disorder hasbeen linked to diseases such as cardiovascular disease, diabetes, gastrointestinal disease, fibromyalgia, chronic fatigue syndrome, and musculoskeletal disorders [17,18]. Research shows that people with long-term post-traumatic stress disorder have higher circulating T lymphocytes and lower cortisol levels, indicating that they are more susceptible to autoimmune diseases. Clinical evidence confirms the association of biological markers with various inflammatory conditions, including cardiovascular and autoimmune diseases [19,20].

Studies highlight that at the cardiovascular system level, agonistic autoantibodies targeting the cardiac β1-adrenergic receptor at the cardiovascular system level are associated with dilated cardiomyopathy [21,22]. Autoantibodies capable of activating α1-adrenergic receptors are associated with refractory hypertension and cardiomyopathy [23,24]. Preeclampsia is characterised by autoantibodies that activate the major angiotensin receptor, AT1 [25,26,27].

Stress can stimulate the production of autoantibodies and autoreactive T-cells, a phenomenon associated with hypertrophic or dilated cardiomyopathies and arrhythmias [25,28]. Activation of macrophages leading to the production and secretion of neuropeptides is linked to depression, anxiety, and fear [29,30].

Acute and chronic psychological stress are now clearly and consistently linked to cardiovascular risk factors, including arrhythmia, heart failure, and insulin resistance and hypertension, respectively, according to expert studies [31,32].

The relationship is ambivalent, since psychological components may be prevalent in some CVDs and predict poorer outcomes, or psychological disorders may be present before CVD develops. A recent cohort study indicated that higher psychosocial stress was substantially linked with mortality as well as CVD, coronary heart disease, and stroke events [33].

In a prospective study, Endrighi et al. showed that long-term psychological stress was a major predictor of cardiovascular morbidity and all-cause mortality in heart failure patients [34]. For instance, several studies have linked high levels of workplace stress to hypertension [35,36].

It has also been demonstrated that post-traumatic stress disorder and other chronic stress disorders are linked to a higher risk of hypertension [37]. Increased chronic psychological stress is independently linked to worse walking capacity and quality of life in individuals with established peripheral artery disease [38].

It is also essential to consider one’s lifestyle habits. Unhealthy habits, such as smoking, limited physical activity, and poor eating habits, cause a significant amount of increased cardiovascular risk linked to stress [39,40].

On the other hand, anxiety is defined as an acute emotional reaction characterised by fleeting fear, uncertainty, and worry about the future, although how frequently and how intensely it affects a person varies [41,42]. Numerous physiological and psychological symptoms, including tachycardia, difficulty relaxing, and terror, are what define it [43].

The relationship between anxiety disorders, deteriorating anxiety symptoms, and coronary heart disease events has been the subject of numerous studies. Anxiety was linked to increased rates of coronary heart disease and cardiovascular death, according to a meta-analysis of 20 trials with follow-up periods longer than 11 years [44].

Several studies have shown that, even after controlling for depression and other conventional risk factors, anxiety remains a risk factor for CVD events [45]. A recent Chinese cohort study found a substantial association between the incidence and mortality of haemorrhagic and ischaemic stroke, as well as acute ischaemic heart disease and panic episodes. For patients diagnosed with generalised anxiety disorder, the risk of incident ischaemic heart disease and haemorrhagic stroke was higher over the follow-up period of ≤6 years [46].

This review identifies and summarises what has been previously published, trying to highlight the effects of stress and anxiety on pregnant women’s cardiovascular health. The purpose of this scoping review is to highlight the impact of stress and anxiety on the cardiovascular health of expectant mothers and to identify the psychometric assessment tools applied for the evaluation of stress and anxiety.

## 2. Methods

### 2.1. Establishing Research Questions

Pregnant women are the target group for this paper, which aims to embrace the most recent data on the relationship between stress and anxiety and cardiovascular health. Therefore, the following research questions were investigated: How do stress and anxiety impact cardiovascular health in pregnant women? How can it be measured? 

### 2.2. Search Techniques for Finding and Choosing Studies

The PRISMA extension for scoping reviews (PRISMA ScR) standards were followed when conducting our scoping review [47]. The review was registered in Inplasy. The registration number is INPLASY2024120081. The search was carried out until the end of 2023, and the following search criteria were applied: articles in English published in the last ten years (since 2014) with the full text available; articles involving human subjects; articles involving adult subjects. An extensive literature search was conducted using the internet databases PubMed, Google Scholar, and Embase. The searches were conducted by three authors with expertise in psychology, psychiatry, and obstetrics and gynaecology, and discrepancies were resolved by consensus (HL, EV, EB). We used different combinations of the following keywords: (‘pregnancy’ OR ‘gestation’) AND (‘psychological stress’ OR ‘anxiety’) AND (‘cardiovascular risk’ OR ’cardiovascular disease’ OR ‘gestational hypertension’ OR ‘pregnancy-induced hypertension’ OR ‘preeclampsia’ OR ‘cardiovascular health’ OR ‘hypertensive disorders of pregnancy’). The search was performed using all possible combinations of these key search terms, but it was not limited to them. Also, all the references from the works included in the review were studied, and studies that were relevant to the subject addressed were identified and included in the review.

The following inclusion criteria were defined: the subjects of the studies must be adult pregnant women, the studies must include the assessment of stress or anxiety through psychometric instruments, and the studies must address the cardiovascular pathologies associated with pregnancy and the impact of stress and/or anxiety. Studies that addressed pregnant women diagnosed with associated psychiatric pathologies at the time of enrolment were excluded.

### 2.3. Extracting and Reporting Data

**Figure 1:**
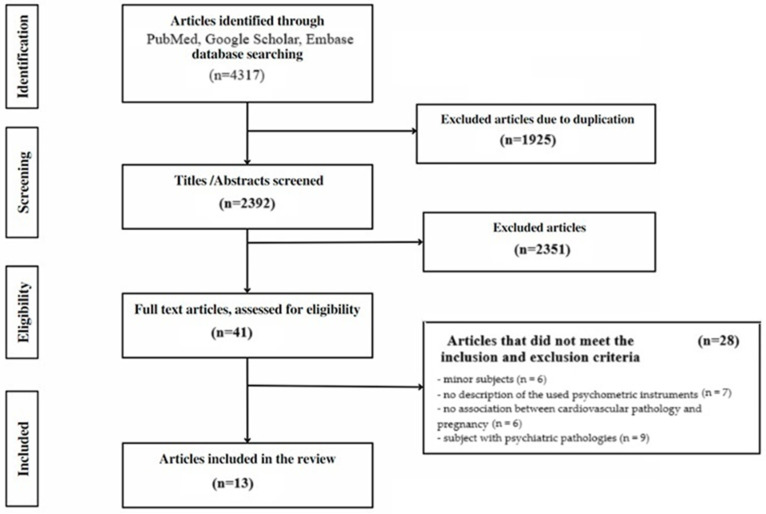
Review process flow chart based on PRISMA ScR principles.

Initially, upon searching the databases, 4317 articles were identified. After eliminating duplicates, the titles and abstracts were separately examined by two reviewers and checked for eligibility. Publications were available only as abstracts, and conference abstracts and study protocols were excluded. A total of 41 publications were identified for possible inclusion and their full texts were retrieved. Articles that did not meet the inclusion and exclusion criteria were removed from the study. Finally, 13 studies with the full text available were used for the review. Each included article was carefully studied in its entirety, and the authors of this study separately extracted pertinent information. Before the articles were finally included, a consensus was established (Figure 1).

### 2.4. Quality Assessment of the Included Studies

For the quality assessment of the studies included in the review, we usedthree risk-of-bias (ROB) instruments: for cohort and case–control studies, we choose the Newcastle–Ottawa Scale (NOS), and for cross-sectional studies, we choose the Agency for Healthcare Research and Quality (AHRQ) test.

The NOS comprises three domains: selection (four items), comparability (one item), and outcome (three items). In a primary study that meets the expected methodological standards, one star is awarded for each item in the selection and outcome domains, with a maximum of two stars allocated to the comparability domain. Research utilising NOS star scores categorised scores of 0 to 4 as a high risk of bias (ROB), 5 to 6 as a moderate ROB, and 7 to 9 as a low ROB. Applying the NOS to nine articles resulted in scores of 7 and 8 for each of them [48]. This means a moderate level of ROB (see Supplementary File, Table S1).

The AHRQ test comprises11 items. If the quality of the study satisfies the methodological requirement, one score is given for each item. A high ROB is indicated by a score of 0–4, a moderate ROB by a score of 5–7, and a low ROB by a score of 8–11 [49]. Applying the AHRQ test to two articles (cohort studies), the scoreswere7 and 8, which indicates a moderate and a low ROB (see Supplementary File, Table S2).

## 3. Results

### StudyCharacteristics

The eligible studies that were selected were published between 2014 and 2023 and included a total of 19,232 participants, with sample sizes ranging from 70 to 8491. Stress was studied in nine studies [50,51,52,53,54,55,56,57,58], while anxiety was studied in 10 articles [49,50,52,53,55,57,58,59,60,61], and depression was also studied in 10 of the articles included in the review [50,53,54,55,57,58,59,60,61,62]. Only in two of the studies is addressed stress [51,56]. In one study, depression was studied in addition to stress [54], and only one study also involved the study of anxiety in addition to stress [52]. A total of five studies used questionnaires that assess stress, anxiety, and depression [50,53,55,57,58]. Four articles included the study of anxiety and depression in parallel [59,60,61,62]. These were assessed using various psychometric questionnaires (Table 1).

The following psychological questionnaires are relevant to the study objective: the Depression Anxiety Stress Scale 21 (DASS-21), Pregnancy-Related Anxiety Questionnaire (PRAQ-R2), Perceived Stress Scale (PSS-10), General Health Questionnaire (GHQ), Spielberger State–Trait Anxiety Inventory (STAI), Perceived Stress Questionnaire (PSQ), Generalised Anxiety Disorder-7 (GAD-7).

The DASS-21 is a self-report scale with 21 items. It evaluates three aspects—stress, anxiety, and depression—each with seven questions. Each item is scored on a Likert scale from 0 (None) to 3 (High). The scores for each scale are calculated by summing all of the specific items. The scores indicate how severe the symptoms are [63].

The PRAQ-R2 is a self-report scale with 10 items that assesses anxiety on three subscales: concern about one’s personal appearance, fear of giving birth, and anxieties about having a child who is physically or mentally disabled [64]. Each item is scored on a Likert scale from 1 (definitely not true) to 5 (definitely true). The PRAQ-R2 total scores vary from 10 to 50, where higher scores correspond to higher anxiety levels.

The PSS-10 is a 10-item self-report questionnaire [65]. It assesses the extent to which a person has in the past month felt that life is unpredictable, out of control, and overwhelming. Using a Likert scale ranging from 0 to 4, two subscales can be computed to evaluate perceived helplessness and perceived self-efficacy in reaction to life experiences.

The GHQ is a self-administered screening questionnaire with a four-point scale: somatic symptoms, anxiety and insomnia, social dysfunction, and severe depression [66]. Participants can select “not at all”, “no more than usual”, “rather more than usual”, and “much more than usual” as their responses on a four-point scale for each item. Scores range from 0 at the lowest to 84 at the highest. Higher distress levels are indicated by higher scores.

The STAI is a 40-item self-report questionnaire with 20 items each that measures trait anxiety (TAI scale) and state anxiety (SAI scale) [67]. State anxiety is thought to be transient and more accurately describes how a person feels when they detect a threat. Trait anxiety is defined as how people feel in everyday events that everyone encounters. For the SAI scale, each item is rated on a four-point Likert scale from 1 to 4, and for the TAI scale, from 0 to 3.

The PSQ is a 30-item self-report questionnaire designed to evaluate stressful life events and situations that often cause or worsen symptoms of disease [68]. On a scale ranging from 1 (meaning “almost never”) to 4 (meaning “usually”), respondents describe how frequently they encounter particular stress-related emotions. Higher stress levels are indicated by higher scores.

The GAD-7 scale was created to evaluate the intensity of symptoms and to identify likely cases of anxiety disorders [69]. There are seven items to rate on a Likert scale, with 0 denoting “not at all” and 3 denoting “almost every day.”

## 4. Discussions

Many people view pregnancy as a happy moment in a woman’s life, filled with happiness, excitement, and other pleasant feelings. However, the future mother’s body experiences hormonal and cardiovascular changes throughout pregnancy, which can make it a vulnerable emotional period and pose problems for her general health. Pregnancy has significant physiological impacts on expectant mothers, including changes in hormones, haemodynamics, and emotions that all impact the cardiovascular system.

### 4.1. Cardiovascular Physiological Adaptative Changes During Pregnancy

The cardiovascular system undergoes significant adaptive physiological changes during pregnancy, intended to help the mother’s body cope with the increased metabolic demands and to ensure adequate maternal–foetal circulation that ensures good development and growth of the foetus [70].

The increase in the levels of oestrogen and progesterone during pregnancy determines, from 5 weeks of gestation, systemic vasodilatation changes that occur before the completion of the placentation process and complete development of the uteroplacental circulation. Studies show that relaxin, a peptide hormone produced by the corpus luteum in pregnancy, could influence small arterial resistance vessels, having an endothelium-dependent effect vasodilator [6,71]. Normally, after birth, after the removal of the placenta, maternal hemodynamic systemic vascular resistance increases, returning to normal in approximately two weeks [72]. These aspects are crucial to understanding the cardiovascular pathology of pregnancy.

Cardiovascular output (CO)must rise to maintain blood pressure, since the placental circulation in pregnancy lacks autoregulation. In the first and second trimesters of pregnancy, cardiac output increases progressively up to 30% compared to the previous pregnancy period, reaching 24 weeks up to an increase of 45%, with a slight decrease near full term [73]. The increase in cardiac output is due to the increase in stroke volume in the first trimester and then to the rise in heart rate, with stroke volume decreasing in the last trimester of pregnancy due to canal compression [74]. Cardiac output changes during pregnancy are presented differently in various studies, which could be explained by the fact that the adaptive changes interfere with the mother’s anthropometric variables and the body’s position [74,75]. During pregnancy, remodelling phenomena of the heart and the entire cardiovascular system are described, intended to support the pregnant woman’s body in adapting to the state of pregnancy.

Thus, the studies note an increase in the thickness and mass of the left ventricle by 28% and 52% [72], respectively, and a 40% increase in the mass of the right ventricle compared to the pre-pregnancy values [76]. Vascular distensibility rises after the systemic vasculature vasodilates [77], and the aortic augmentation index, a measure of aortic stiffness, falls sharply from the start of pregnancy and reaches its lowest point in the second trimester as a result of the load gradually increasing in the final trimester [78,79].

Normal pregnancy’s physiological changes can cause symptoms and indicators that are sometimes mistaken for heart disease, making it more difficult to diagnose CVD, particularly when no risk factors for the condition have been found. These symptoms include severe or progressive dyspnoea, paroxysmal nocturnal dyspnoea, progressive orthopnoea, nausea and heartburn, epigastric or chest pain (gastro-oesophageal reflux), precordial pain on effort or after emotions, syncope, haemoptysis, peripheral oedema, distended neck veins, lateral displacement of the cardiac apex, and the presence of a third heart sound and ejection systolic murmur [80,81,82,83,84].

Prenatal and postpartum cardiovascular disease screenings should be performed on all women in order to identify women who may be at risk and to inform and increase awareness among patients and healthcare professionals about the differences between adaptive physiological changes and pathological conditions related to cardiovascular health. This tool could contribute to reducing CVD morbidity and mortality among pregnant women [6].

### 4.2. Impact of Stress and Anxiety on Cardiovascular Health in Pregnant Women

Pregnancy-related cardiac disease is associated with high rates of morbidity and hospitalisation. Heart disease during pregnancy is linked to an increased risk of eclampsia, caesarean delivery, and postpartum haemorrhage; about one in four pregnant women with cardiac disease are admitted to the hospital throughout their pregnancy [1].

The studies highlight that, during pregnancy and in the period immediately following birth, a broad spectrum of heart diseases are diagnosed, both from the category of congenital ones [85,86,87] and those acquired before the woman becomes pregnant or during pregnancy, such as rheumatic heart disease [86,88,89].

The relationship between stress, anxiety, and cardiovascular health in pregnant women is complex and involves various physiological and psychological mechanisms. This section synthesises findings from multiple studies to provide a comprehensive overview of how stress and anxiety affect cardiovascular health in pregnant women.

Thombre et al. (2015) found that pre-pregnancy depression and anxiety symptoms significantly increase the risk of hypertensive disorders, particularly chronic hypertension (CH) and preeclampsia with preterm delivery. The timing of these symptoms is crucial, with pre-pregnancy symptoms having a stronger association with hypertensive disorders than those occurring during pregnancy [61].

Similarly, anxiety during pregnancy is a major predictor of hypertension, according to Nath et al. (2021). Compared to women who were not nervous, anxious women were more likely to acquire hypertension. According to this study, women who have a history of elevated blood pressure during pregnancy are more likely to develop cardiovascular diseases (CVDs) in the future because of common risk factors for CVD, including chronic hypertension and type 2 diabetes [62].

Garza-Veloz et al. (2017) further highlighted the effects of stress by discovering a strong correlation between maternal anxiety and the emergence of pregnancy-related hypertensive illnesses, such as gestational hypertension (GH) and preeclampsia (PE). Hypertensive disorder of pregnancy (HDP) was more likely to occur in women with anxiety, sleeplessness, social dysfunction, and severe physical symptoms than in those without these symptoms [52].

Adding to this body of evidence, Kordi et al. (2017) discovered a significant relationship between anxiety and the incidence of preeclampsia. Women with anxiety had a 2.90-fold increased risk of developing preeclampsia. This study suggested that psychological stress and anxiety could lead to increased levels of proinflammatory cytokines and stress-induced changes in the sympathetic nervous system, contributing to the development of preeclampsia [53].

Horsley et al. (2022) found that depressive symptoms and state anxiety exacerbate the negative impact of HDP on gestational age. The relationship between HDP and shorter gestational duration was more pronounced for women who experienced higher levels of anxiety and depression symptoms [60].

Furthermore, Ardani et al. (2020) assessed affective temperaments in pregnant women and found that anxious temperament scores were significantly higher in women with GH compared to normotensive controls. This suggests that anxious temperament, reflecting heightened anxiety and stress levels, is associated with the development of gestational hypertension and other cardiovascular risk factors like depressive temperament [58].

Anxiety and depression symptoms during pregnancy have different effects on maternal–foetal vascular function, as noted by Bilbul et al. (2022). Early pregnancy anxieties of childbirth were linked to decreased resistance to umbilical blood flow in mid-to-late pregnancy, suggesting a possible effect on foetal–placental development. On the other hand, lower systolic and diastolic blood pressure (SBP and DBP) in the early stages of pregnancy were linked to higher intensity of maternal depressive symptoms. This mediated the association between a lower chance of developing HDP later on and more substantial depressive symptoms in mothers [59].

Lackner et al. (2018) found that both chronic stress and a history of preeclampsia independently contribute to blunted cardiac responses to acute psychological challenges. Women with higher chronic stress levels and those with a history of preeclampsia exhibited smaller heart rate responses to stressors, indicating an inability to mobilise an appropriate heart rate response to stress, which is crucial for effective coping. This reduced cardiac responsiveness is linked to negative health outcomes and suggests a higher chance of developing cardiovascular problems in the future [54].

In line with these findings, Lanssens et al. (2023) observed that levels of anxiety and depression significantly increased from the time of enrolment (around 14 weeks of gestation) to 32 weeks of gestation. According to the study, pregnant women who are at risk for pregnancy-induced hypertension (PIH) deal with increased levels of anxiety and depression as their pregnancy goes on, which can have a negative impact on their cardiovascular health. Elevated anxiety and depression can lead to higher blood pressure and other cardiovascular issues, complicating the management of PIH [55].

Boyer et al. (2023) emphasised the multidimensional nature of psychosocial stress and its association with suboptimal cardiovascular health (CVH) during pregnancy. According to the study, women in the most disadvantaged psychosocial stressor class had a roughly threefold higher likelihood of having deficient CVH than those in the most advantaged class. The study also identified different classes of psychosocial stressors. Behavioural factors such as inadequate physical activity, obesity, and smoking were significant drivers of suboptimal CVH, suggesting that stress and anxiety may contribute to unhealthy behaviours that negatively impact cardiovascular health [50].

Additionally, Parisi et al. (2024) examined the impact of stress and anxiety on heart rate variability (HRV) in pregnant women, finding that higher levels of perceived parenting stress were associated with lower HRV. This indicates reduced cardiac flexibility and increased cardiovascular risk. The study found no significant correlation between maternal HRV and infant HRV, suggesting that the relationship between stress and cardiovascular health is more directly related to the mother’s physiological state [57].

Extending the scope of stress-related cardiovascular impacts, Oni et al. (2015) explored the effects of maternal stress and anxiety on maternal–foetal circulation. The study suggested that maternal stress, particularly anxiety, adversely affects maternal–foetal circulation by co-activating the maternal sympathetic nervous system and increasing foetal catecholamines. This not only impacts the cardiovascular system but also impairs neurodevelopment. The intergenerational impact of stress and anxiety is highlighted by the possibility that severe maternal stress and early and mid-gestational exposure to glucocorticoids may raise the offspring’s risk of cardiovascular diseases [56].

Monk et al. (2020) added a long-term perspective, highlighting the prolonged impact of prenatal stress on cardiovascular health. The study found that higher levels of perceived stress during the first trimester and increases in stress between the first and third trimesters were associated with higher stress levels 2–7 years after delivery. Adverse pregnancy outcomes such as HDP and preeclampsia were independently associated with higher stress levels years after pregnancy, which may contribute to future cardiovascular health risks. This underscores the lasting influence of prenatal stress on women’s health well beyond pregnancy [51].

The studies included in this review use several psychometric tools to assess stress or anxiety. Also, the cardiovascular parameters measured are not the same in all studies. Therefore, the results obtained by applying various scales used in psychometric assessments, such as DASS-21 versus STAI, may influence the reported prevalence of anxiety and its cardiovascular impact.

All the studies demonstrated the association of stress and anxiety during pregnancy with cardiovascular pathologies. Hypertension induced by pregnancy and its complication—preeclampsia—were studied in most works (69%) [51,52,53,54,55,58,60,61,62]. The findings of every study provided evidence for the clear connection between anxiety and stress and the development of this cardiovascular disease during pregnancy.

All studies emphasise the significance of addressing psychosocial stress and anxiety during pregnancy in a broader context of factors, including social determinants, as they may have an impact on the mother’s health as well as the course of the pregnancy, regardless of the psychological instrument used. The findings from these studies may be applied in clinical practice to develop psychometric screening tools for use during pregnancy.

The prevalence of cardiovascular risk factors, particularly obesity, diabetes, and hypertension, rises with a woman’s age when she becomes pregnant for the first time, potentially increasing the number of instances of CVD during pregnancy [90]. For the early detection of pregnant women with the potential to develop CVD, it is imperative to know and identify the factors that could constitute a cardiovascular risk. Preventing CVD in pregnant women and applying prompt measures to cases diagnosed with CVD could lead to a decrease in the number of complications and maternal deaths from this cause [1]. Long-term psychosocial stress raises the risk of CVD by the same amount as traditional risk factors [91].

The evaluation of the physical and psychological health of pregnant women during periodic prenatal consultations can contribute to the early diagnosis of stress and anxiety, which can impact the cardiovascular health of the pregnant woman. Routine screening for anxiety and depressive symptoms during prenatal care, using validated instruments, is crucial. Every pregnant woman should be assessed for stress and anxiety using standardised, validated tools, according to the American College of Obstetricians and Gynaecologists’ clinical practice guideline on the screening and diagnosis of mental health conditions during pregnancy and postpartum [92].

The guidelines do not recommend routine screening for cardiovascular diseases in pregnancy. However, since the importance of detecting these pathologies as early as possible is known, any symptomatology that would suggest a cardiovascular disease must be investigated [93]. Therefore, it is essential to evaluate the cardiovascular risk of the pregnant woman at the time of the first visit and then throughout the entire period of pregnancy. Although stress and anxiety can impact the cardiovascular health of expectant mothers, there are no guidelines for detecting stress and anxiety in pregnant women combined with screening for cardiovascular diseases.

Applying standardised, validated psychological instruments to evaluate pregnant women for stress and anxiety as early as possible in pregnancy, preferably when the pregnancy is taken into account, could have important clinical implications. The detection of these cases would facilitate the application of potential interventions to reduce stress and, thus, could also mitigate the cardiovascular risk in the pregnant women in question. Therefore, in such cases, interdisciplinary approaches are necessary: an obstetrician would monitor the pregnancy and apply the specific tests to identify stress and anxiety, and a psychologist would interpret the test results and, in a situation where a patient is identified as experiencing stress or anxiety, could notify an obstetrician, who could refer the case to a cardiologist for evaluation. Providing psychological support and stress management interventions, such as cognitive–behavioural therapy, could potentially mitigate the adverse effects of stress and anxiety on cardiovascular health. Early intervention and continuous monitoring of cardiovascular risk factors in women with a history of hypertensive disorders or high stress levels are recommended to improve their long-term health outcomes. Comprehensive prenatal care that includes mental health evaluations and interventions is essential to address these risks and improve overall health outcomes for pregnant women and their offspring. By linking the various aspects of hypertensive disorders, heart rate variability, maternal–foetal circulation, and long-term health, the interconnected nature of stress and anxiety’s impact becomes apparent, emphasising the need for holistic and integrated prenatal care strategies.

Considering the association of the two medical conditions, it is recommended that women in whom the presence of stress or anxiety has been demonstrated also be evaluated from a cardiovascular point of view. Thus, these pathologies could be diagnosed early and would offer the possibility of prompt medical interventions that could lead to a decrease in morbidity and mortality from cardiovascular diseases.

## 5. Limitations

This review presents limitations in terms of objectivity. Firstly, the search was limited to three databases from specialised literature. Also, different articles used more psychometric questionnaires to detect stress and anxiety. In addition, there were different sample sizes and different methodological approaches. Most study sample sizes were small, and the questionnaires used in the studies were self-reported, so there may be over- and under-reporting. Because of all of this, estimating the effect of stress on pregnant women’s cardiovascular health is challenging. In addition, only English-language keywords and articles published in or translated into English were included in the search parameters. More studies are needed to clarify which are the best tools for assessing stress and anxiety in pregnancy and their effects on cardiovascular health in pregnant women.

## 6. Conclusions

The cardiovascular health of a pregnant woman is greatly impacted by stress and anxiety. An interdisciplinary approach is necessary when treating an expectant mother who has been found to be experiencing stress and anxiety in addition to pathological cardiovascular conditions. Understanding and differentiating between the physiological changes that the body undergoes during pregnancy due to hormonal fluctuations and the cardiovascular pathology that is impacted by the patient’s stress and worry is crucial for appropriate care.

Screening for psychological and cardiovascular risk factors performed before or during the pregnancy could help identify cases that require specific medical and psychological interventions, which can contribute to decreasing maternal morbidity and mortality related to CVD.

These factors facilitate the implementation of effective clinical practice programmes that promptly identify patients at risk of developing cardiovascular pathologies during pregnancy and apply appropriate intervention measures. Politicians and professionals should advocate for the implementation of a pregnancy monitoring methodology which requires pregnant women to complete specific questionnaires that may reveal the presence of stress and anxiety.

The existence of longitudinal studies and policy initiatives for systematic screening demonstrates concerns related to this subject.

Future research is essential to systematically screen for stress and anxiety during the first trimester of pregnancy, focusing on the implementation of psychological interventions and assessing their impact on the incidence of cardiovascular pathology. Also, these studies would help us develop a better understanding of the role of maternal stress in the development of cardiovascular disorders in pregnant women.

## Figures and Tables

**Table 1:** This list of the screened articles illustrates the types of studies, years of publication, sample size, psychometric tests used, and investigated cardiovascular diseases.

Authors	Years	Study Type	Sample Size	Type of Distress	Psychometric Questionnaire	Studied Cardiovascular Parameters/Diseases
Ardani et al. [58]	2020	Cross-sectional	N = 240	stress, anxiety, depression	TEMPS-A, DASS 21	GH
Bilbul et al. [59]	2022	Prospective cohort	N = 406	anxiety, depression	EPDS in the first trimester, PRAQ-R2 in the third trimester	maternal–foetal vascular function
Boyer et al. [50]	2023	Prospective cohort	N = 8491	stress, anxiety, depression	EPDS, STAI-AD, PSS-10	general cardiovascular health
Garza-Volez et al. [52]	2017	Prospective cohort	N = 321	stress, anxiety	GHQ	HDP, including PE and GH
Horsley et al. [60]	2022	Prospective cohort	N = 2763	anxiety, depression	EPDS, SAI	HDP
Kordi et al. [53]	2017	Case–control	N = 150	stress, anxiety, depression	DASS-21	PE
Lackner et al. [54]	2018	Prospective cohort	N = 70	stress, depression	PSQ, ADS	PE
Lanssens et al. [55]	2023	Observational	N = 110	stress, anxiety, depression	GAD-7, PHQ-9, PCS	GH
Monk et al. [51]	2020	Prospective cohort	N = 4161	stress	PSS-10	HDP-PE
Nath et al. [62]	2021	Prospective cohort	N = 783	anxiety, depression	EPDS, PRA, MSPSS, Revised Kuppuswamy Scale, RDAS, MCTS	hypertensive disorders
Oni et al. [56]	2015	Cross-sectional	N = 146	stress	PSS, Carver’s Brief COPE	general cardiovascular health
Parisi et al. [57]	2024	Observational	N = 220	stress, anxiety, depression	PRAQ, EPDS, PTQ, PSI, MPAS	heart rate variability
Thombre et al. [61]	2015	Prospective cohort	N = 1371	anxiety, depression	CES-D	hypertensive disorders

Abbreviations: TEMPS-A—Temperament Evaluation of Memphis; DASS 21—Depression, Anxiety, and Stress Scale; GH—gestational hypertension; EPDS—Edinburgh Postnatal Depression Scale; PRAQ-R2, Pregnancy-Related Anxiety Questionnaire; STAI-AD, State–Trait Anxiety Inventory for Adults; PSS-10—Perceived Stress Scale; GHQ, General Health Questionnaire; HDP—hypertensive disorders of pregnancy; PE—preeclampsia; SAI—the state anxiety subscale of the Spielberger State–Trait Anxiety Inventory; PSQ, Perceived Stress Questionnaire; ADS—Allgemeine Depression Scale; GAD-7—Generalised Anxiety Disorder-7 Scale; PHQ-9—Patient Health Questionnaire-9; PCS—Pain Catastrophising Scale; PRA—Pregnancy-Related Anxiety Scale; MSPSS—Multidimensional Scale of Perceived Social Support; RDAS—Revised Dyadic Adjustment Scale; MCTS—Modified Conflict Tactics Scale; PSS—Cohen Perceived Stress Scale; PRAQ—Pregnancy-Related Anxiety Questionnaire—Revised; PTQ—Perseverative Thinking Questionnaire; PSI—Parent Stress Index; MPAS—Maternal Postnatal Attachment Scale; CES-D—Center for Epidemiologic Study Depression Scale.

## Data Availability

Not applicable.

## Supplementary Materials

The following supporting information can be downloaded at: https://www.mdpi.com/article/10.3390/jcm14030909/s1, Table S1: Quality assessment of cohort and case-control studies using Newcastle-Ottawa Scale; Table S2: Quality assessment of the cross-sectional studies evaluated by AHRQ.

## References

[1] Roos-Hesselink J.W. (2018). The Task Force for the Management of Cardiovascular Diseases during Pregnancy of the European Society of Cardiology (ESC). Eur. Heart J..

[2] Tsao C.W., Aday A.W., Almarzooq Z.I., Alonso A., Beaton A.Z., Bittencourt M.S., Boehme A.K., Buxton A.E., Carson A.P., Commodore-Mensah Y. (2022). Heart Disease and Stroke Statistics—2022 Update: A Report from the American Heart Association. Circulation.

[3] Cushman M., Shay C.M., Howard V.J., Jiménez M.C., Lewey J., McSweeney J.C., Newby L.K., Poudel R., Reynolds H.R., Rexrode K.M. (2021). Ten-Year Differences in Women’s Awareness Related to Coronary Heart Disease: Results of the 2019 American Heart Association National Survey: A Special Report from the American Heart Association. Circulation.

[4] Shufelt C.L., Pacheco C., Tweet M.S., Miller V.M., Kerkhof P.L.M., Miller V.M. (2018). Sex-Specific Physiology and Cardiovascular Disease. Sex-Specific Analysis of Cardiovascular Function.

[5] Elkayam U., Goland S., Pieper P.G., Silversides C.K. (2016). High-Risk Cardiac Disease in Pregnancy. J. Am. Coll. Cardiol..

[6] Chambers M.E., De Zoysa M.Y., Hameed A.B. (2022). Screening for Cardiovascular Disease in Pregnancy: Is There a Need?. J. Cardiovasc. Dev. Dis..

[7] Gongora M., Wenger N. (2015). Cardiovascular Complications of Pregnancy. Int. J. Mol. Sci..

[8] Wang W., Lin R., Yang L., Wang Y., Mao B., Xu X., Yu J. (2023). Meta-Analysis of Cardiovascular Risk Factors in Offspring of Preeclampsia Pregnancies. Diagnostics.

[9] Selye H. (1978). The Stress of Life.

[10] Schneiderman N., Ironson G., Siegel S.D. (2005). Stress and Health: Psychological, Behavioral, and Biological Determinants. Annu. Rev. Clin. Psychol..

[11] Leger K.A., Charles S.T., Almeida D.M. (2020). Positive Emotions Experienced on Days of Stress Are Associated with Less Same-Day and Next-Day Negative Emotion. Affect. Sci..

[12] Yaribeygi H., Panahi Y., Sahraei H., Johnston T.P., Sahebkar A. (2017). The Impact of Stress on Body Function: A Review. EXCLI J..

[13] Jalal S.M., Alsebeiy S.H., Alshealah N.M.J. (2024). Stress, Anxiety, and Depression During Pregnancy: A Survey Among Antenatal Women Attending Primary Health Centers. Healthcare.

[14] Ménard C., Pfau M.L., Hodes G.E., Russo S.J. (2017). Immune and Neuroendocrine Mechanisms of Stress Vulnerability and Resilience. Neuropsychopharmacology.

[15] Henein M.Y., Vancheri S., Longo G., Vancheri F. (2022). The Impact of Mental Stress on Cardiovascular Health—Part II. J. Clin. Med..

[16] Vancheri F., Longo G., Vancheri E., Henein M.Y. (2022). Mental Stress and Cardiovascular Health—Part I. J. Clin. Med..

[17] Black P.H. (2003). The Inflammatory Response Is an Integral Part of the Stress Response: Implications for Atherosclerosis, Insulin Resistance, Type II Diabetes and Metabolic Syndrome X. Brain Behav. Immun..

[18] Stojanovich L., Marisavljevich D. (2008). Stress as a Trigger of Autoimmune Disease. Autoimmun. Rev..

[19] Boscarino J.A. (2004). Posttraumatic Stress Disorder and Physical Illness: Results from Clinical and Epidemiologic Studies. Ann. N. Y. Acad. Sci..

[20] Kozora E., Ellison M.C., Waxmonsky J.A., Wamboldt F.S., Patterson T.L. (2005). Major Life Stress, Coping Styles, and Social Support in Relation to Psychological Distress in Patients with Systemic Lupus Erythematosus. Lupus.

[21] Jahns R., Boivin V., Lohse M.J. (2006). B1-Adrenergic Receptor Function, Autoimmunity, and Pathogenesis of Dilated Cardiomyopathy. Trends Cardiovasc. Med..

[22] Nikolaev V.O., Boivin V., Störk S., Angermann C.E., Ertl G., Lohse M.J., Jahns R. (2007). A Novel Fluorescence Method for the Rapid Detection of Functional Β1-Adrenergic Receptor Autoantibodies in Heart Failure. J. Am. Coll. Cardiol..

[23] Wenzel K., Haase H., Wallukat G., Derer W., Bartel S., Homuth V., Herse F., Hubner N., Schulz H., Janczikowski M. (2008). Potential Relevance of A1-Adrenergic Receptor Autoantibodies in Refractory Hypertension. PLoS ONE.

[24] Yan L., Xu Y., Yao H., Xue W., Tian J., Ren H., Wu Y., Yang G., Ma X.L., Liu H. (2009). The Effects of Autoantibodies against the Second Extracellular Loop of A1-Adrenoceptor on Vasoconstriction. Basic Res. Cardiol..

[25] Xia Y., Zhou C.C., Ramin S.M., Kellems R.E. (2007). Angiotensin Receptors, Autoimmunity, and Preeclampsia. J. Immunol..

[26] Xia Y., Kellems R.E. (2009). Is Preeclampsia an Autoimmune Disease?. Clin. Immunol..

[27] Yang X., Wang F., Chang H., Zhang S., Yang L., Wang X., Cheng X., Zhang M., Ma X.L., Liu H. (2008). Autoantibody against AT1 Receptor from Preeclamptic Patients Induces Vasoconstriction through Angiotensin Receptor Activation. J. Hypertens..

[28] Wang B., Hu S., Shi D., Bing Z., Li Z. (2019). Arrhythmia and/or Cardiomyopathy Related to Maternal Autoantibodies: Descriptive Analysis of a Series of 16 Cases from a Single Center. Front. Pediatr..

[29] Dye C., Lenz K.M., Leuner B. (2022). Immune System Alterations and Postpartum Mental Illness: Evidence from Basic and Clinical Research. Front. Glob. Womens Health.

[30] Ravi M., Bernabe B., Michopoulos V. (2022). Stress-Related Mental Health Disorders and Inflammation in Pregnancy: The Current Landscape and the Need for Further Investigation. Front. Psychiatry.

[31] Havranek E.P., Mujahid M.S., Barr D.A., Blair I.V., Cohen M.S., Cruz-Flores S., Davey-Smith G., Dennison-Himmelfarb C.R., Lauer M.S., Lockwood D.W. (2015). Social Determinants of Risk and Outcomes for Cardiovascular Disease: A Scientific Statement from the American Heart Association. Circulation.

[32] Rejack G., Brown A., Nicholls S., Keage H. (2019). Associations Between Cardiovascular Burden, Gender and Depression in Indigenous Australians. Heart Lung Circ..

[33] Santosa A., Rosengren A., Ramasundarahettige C., Rangarajan S., Gulec S., Chifamba J., Lear S.A., Poirier P., Yeates K.E., Yusuf R. (2021). Psychosocial Risk Factors and Cardiovascular Disease and Death in a Population-Based Cohort from 21 Low-, Middle-, and High-Income Countries. JAMA Netw. Open.

[34] Endrighi R., Dimond A.J., Waters A.J., Dimond C.C., Harris K.M., Gottlieb S.S., Krantz D.S. (2019). Associations of Perceived Stress and State Anger with Symptom Burden and Functional Status in Patients with Heart Failure. Psychol. Health.

[35] Guimont C., Brisson C., Dagenais G.R., Milot A., Vézina M., Mâsse B., Moisan J., Laflamme N., Blanchette C. (2006). Effects of Job Strain on Blood Pressure: A Prospective Study of Male and Female White-Collar Workers. Am. J. Public Health.

[36] Markovitz J.H., Matthews K.A., Whooley M., Lewis C.E., Greenlund K.J. (2004). Increases in Job Strain Are Associated with Incident Hypertension in the CARDIA Study. Ann. Behav. Med..

[37] Kibler J.L., Joshi K., Ma M. (2009). Hypertension in Relation to Posttraumatic Stress Disorder and Depression in the US National Comorbidity Survey. Behav. Med..

[38] Aquarius A.E. (2006). Clinical Indicators and Psychosocial Aspects in Peripheral Arterial Disease. Arch. Surg..

[39] Hamer M., Molloy G.J., Stamatakis E. (2008). Psychological Distress as a Risk Factor for Cardiovascular Events. J. Am. Coll. Cardiol..

[40] Lallukka T., Lahelma E., Rahkonen O., Roos E., Laaksonen E., Martikainen P., Head J., Brunner E., Mosdol A., Marmot M. (2008). Associations of Job Strain and Working Overtime with Adverse Health Behaviors and Obesity: Evidence from the Whitehall II Study, Helsinki Health Study, and the Japanese Civil Servants Study. Soc. Sci. Med..

[41] American Psychiatric Association (2013). Diagnostic and Statistical Manual of Mental Disorders.

[42] Barlow D. (2004). Anxiety and Its Disorders: The Nature and Treatment of Anxiety and Panic.

[43] Steer R., Beck A. (1997). Beck Anxiety Inventory. Evaluating Stress: A Book of Resources.

[44] Roest A.M., Martens E.J., De Jonge P., Denollet J. (2010). Anxiety and Risk of Incident Coronary Heart Disease. J. Am. Coll. Cardiol..

[45] Stewart J.C., Hawkins M.A.W., Khambaty T., Perkins A.J., Callahan C.M. (2016). Depression and Anxiety Screens as Predictors of 8-Year Incidence of Myocardial Infarction and Stroke in Primary Care Patients. Psychosom. Med..

[46] Wu M., Zhu Y., Lv J., Guo Y., Yang L., Chen Y., Tang W., Xiang S., Sun X., Chen J. (2022). Association of Anxiety with Cardiovascular Disease in a Chinese Cohort of 0.5 Million Adults. J. Affect. Disord..

[47] Tricco A.C., Lillie E., Zarin W., O’Brien K.K., Colquhoun H., Levac D., Moher D., Peters M.D.J., Horsley T., Weeks L. (2018). PRISMA Extension for Scoping Reviews (PRISMA-ScR): Checklist and Explanation. Ann. Intern. Med..

[48] Wells G.A., Shea B., O’Connell D.A., Peterson J., Welch V., Losos M., Tugwell P. (2000). The Newcastle-Ottawa Scale (NOS) for Assessing the Quality of Nonrandomised Studies in Meta-Analyses, Oxford, UK. https://www.ohri.ca/programs/clinical_epidemiology/oxford.asp.

[49] Viswanathan M., Patnode C.D., Berkman N.D., Bass E.B., Chang S., Hartling L., Murad M.H., Treadwell J.R., Kane R.L. (2017). Assessing the Risk of Bias of Individual Studies in Systematic Reviews of Health Care Interventions.

[50] Boyer T.M., Avula V., Minhas A.S., Vaught A.J., Sharma G., Gemmill A. (2023). Psychosocial Stressors as a Determinant of Maternal Cardiovascular Health During Pregnancy. Am. J. Cardiol..

[51] Monk C., Webster R.S., McNeil R.B., Parker C.B., Catov J.M., Greenland P., Bairey Merz C.N., Silver R.M., Simhan H.N., Ehrenthal D.B. (2020). Associations of Perceived Prenatal Stress and Adverse Pregnancy Outcomes with Perceived Stress Years after Delivery. Arch. Womens Ment. Health.

[52] Garza-Veloz I., Castruita-De La Rosa C., Ortiz-Castro Y., Flores-Morales V., Castañeda-Lopez M.E., Cardenas-Vargas E., Hernandez-Delgadillo G.P., Ortega-Cisneros V., Luevano M., Rodriguez-Sanchez I.P. (2017). Maternal Distress and the Development of Hypertensive Disorders of Pregnancy. J. Obstet. Gynaecol..

[53] Kordi M., Vahed A., Rezaee T., Mazloum S., Lotfalizadeh M. (2017). Anxiety during Pregnancy and Preeclampsia: A Case-Control Study. J. Midwifery Reprod. Health.

[54] Lackner H.K., Moertl M.G., Schmid-Zalaudek K., Lucovnik M., Weiss E.M., Kolovetsiou-Kreiner V., Papousek I. (2018). History of Preeclampsia Adds to the Deleterious Effect of Chronic Stress on the Cardiac Ability to Flexibly Adapt to Challenge. Front. Physiol..

[55] Lanssens D., Vandenberk T., Storms V., Thijs I., Grieten L., Bamelis L., Gyselaers W., Tang E., Luyten P. (2023). Changes in Intrapersonal Factors of Participants in the Pregnancy Remote Monitoring Study Who Are at Risk for Pregnancy-Induced Hypertension: Descriptive Quantitative Study. J. Med. Internet Res..

[56] Oni O., Harville E., Xiong X., Buekens P. (2015). Relationships Among Stress Coping Styles and Pregnancy Complications Among Women Exposed to Hurricane Katrina. J. Obstet. Gynecol. Neonatal Nurs..

[57] Parisi F., Høifødt R.S., Bohne A., Wang C.E.A., Pfuhl G. (2024). Perceived Parenting Stress Is Related to Cardiac Flexibility in Mothers: Data from the NorBaby Study. Behav. Sci..

[58] Rezaei Ardani A., Tara F., Naghizadeh Kashani S., Hatami S.B., Emadzadeh M., Nahidi M. (2020). Is Gestational Hypertension Associated with Affective Temperaments?. Hypertens. Pregnancy.

[59] Bilbul M., Caccese C., Horsley K., Gauvreau A., Gavanski I., Montreuil T., Konci R., Lai J.K., Da Costa D., Zelkowitz P. (2022). Maternal Anxiety, Depression and Vascular Function during Pregnancy. J. Psychosom. Res..

[60] Horsley K., Ramsay J.O., Mennitto S., Ditto B., Da Costa D. (2022). Maternal Cardiovascular Risk Factors, Symptoms of Depression and Pregnancy-Specific Anxiety, and Blood Pressure Across Gestation: A Functional Data Analytic Approach. Psychosom. Med..

[61] Thombre M.K., Talge N.M., Holzman C. (2015). Association Between Pre-Pregnancy Depression/Anxiety Symptoms and Hypertensive Disorders of Pregnancy. J. Women’s Health.

[62] Nath A., B S., Raj S., Metgud C.S. (2021). Prevalence of Hypertension in Pregnancy and Its Associated Factors among Women Attending Antenatal Clinics in Bengaluru. J. Fam. Med. Prim. Care.

[63] Lovibond S.H., Lovibond P.F. (1996). Manual for the Depression Anxiety Stress Scales.

[64] Huizink A.C., Delforterie M.J., Scheinin N.M., Tolvanen M., Karlsson L., Karlsson H. (2016). Adaption of Pregnancy Anxiety Questionnaire–Revised for All Pregnant Women Regardless of Parity: PRAQ-R2. Arch. Womens Ment. Health.

[65] Cohen S., Kamarck T., Mermelstein R. (1983). A Global Measure of Perceived Stress. J. Health Soc. Behav..

[66] Goldberg D.P., Hillier V.F. (1979). A Scaled Version of the General Health Questionnaire. Psychol. Med..

[67] Spielberger C.D., Gorsuch R.L. (1983). Manual for the State-Trait Anxiety Inventory (Form Y).

[68] Levenstein S., Prantera C., Varvo V., Scribano M.L., Berto E., Luzi C., Andreoli A. (1993). Development of the Perceived Stress Questionnaire: A New Tool for Psychosomatic Research. J. Psychosom. Res..

[69] Spitzer R.L., Kroenke K., Williams J.B.W., Löwe B. (2006). A Brief Measure for Assessing Generalized Anxiety Disorder: The GAD-7. Arch. Intern. Med..

[70] Metcalfe J., Ueland K. (1974). Maternal Cardiovascular Adjustments to Pregnancy. Prog. Cardiovasc. Dis..

[71] Fisher C., MacLean M., Morecroft I., Seed A., Johnston F., Hillier C., McMurray J. (2002). Is the Pregnancy Hormone Relaxin Also a Vasodilator Peptide Secreted by the Heart?. Circulation.

[72] Robson S.C., Dunlop W., Moore M., Hunter S. (1987). Haemodynamic Changes during the Puerperium: A Doppler and M-mode Echocardiographic Study. BJOG Int. J. Obstet. Gynaecol..

[73] Ueland K. (1976). Maternal Cardiovascular Dynamics. Am. J. Obstet. Gynecol..

[74] Adeyeye V.O., Balogun M.O., Adebayo R.A., Makinde O.N., Akinwusi P.O., Ajayi E.A., Ogunyemi S.A., Akintomide A.O., Ajayi E.O., Adeyeye A.G. (2016). Echocardiographic Assessment of Cardiac Changes during Normal Pregnancy among Nigerians. Clin. Med. Insights Cardiol..

[75] Sanghavi M., Rutherford J.D. (2014). Cardiovascular Physiology of Pregnancy. Circulation.

[76] Ducas R.A., Elliott J.E., Melnyk S.F., Premecz S., daSilva M., Cleverley K., Wtorek P., Mackenzie G.S., Helewa M.E., Jassal D.S. (2014). Cardiovascular Magnetic Resonance in Pregnancy: Insights from the Cardiac Hemodynamic Imaging and Remodeling in Pregnancy (CHIRP) Study. J. Cardiovasc. Magn. Reson..

[77] Poppas A., Shroff S.G., Korcarz C.E., Hibbard J.U., Berger D.S., Lindheimer M.D., Lang R.M. (1997). Serial Assessment of the Cardiovascular System in Normal Pregnancy: Role of Arterial Compliance and Pulsatile Arterial Load. Circulation.

[78] Fujime M., Tomimatsu T., Okaue Y., Koyama S., Kanagawa T., Taniguchi T., Kimura T. (2012). Central Aortic Blood Pressure and Augmentation Index during Normal Pregnancy. Hypertens. Res..

[79] Mahendru A.A., Everett T.R., Wilkinson I.B., Lees C.C., McEniery C.M. (2014). A Longitudinal Study of Maternal Cardiovascular Function from Preconception to the Postpartum Period. J. Hypertens..

[80] Bondagji N.S. (2012). Ischaemic Heart Disease in Pregnancy. J. Saudi Heart Assoc..

[81] Choi H.S., Han S.S., Choi H.A., Kim H.S., Lee C.G., Kim Y.Y., Hwang J.J., Park J.B., Shin H.H. (2001). Dyspnea and Palpitation during Pregnancy. Korean J. Intern. Med..

[82] García-Rio F., Pino J.M., Gómez L., Alvarez-Sala R., Villasante C., Villamor J. (1996). Regulation of Breathing and Perception of Dyspnea in Healthy Pregnant Women. Chest.

[83] Hubbard W.N., Jenkins B.A., Ward D.E. (1983). Persistent Atrial Tachycardia in Pregnancy. Br. Med. J..

[84] Milne J.A., Howie A.D., Pack A.I. (1978). Dyspnoea During Normal Pregnancy. BJOG Int. J. Obstet. Gynaecol..

[85] Claessens P., Moons P., De Casterlé B.D., Cannaerts N., Budts W., Gewillig M. (2005). What Does It Mean to Live with a Congenital Heart Disease? A Qualitative Study on the Lived Experiences of Adult Patients. Eur. J. Cardiovasc. Nurs..

[86] Gantt L.T. (1992). Growing up Heartsick: The Experiences of Young Women with Congenital Heart Disease. Health Care Women Int..

[87] Ngu K., Hay M., Menahem S. (2014). Case Studies of the Perceptions of Women with High Risk Congenital Heart Disease Successfully Completing a Pregnancy. Heart Lung Circ..

[88] Moons P., Bovijn L., Budts W., Belmans A., Gewillig M. (2010). Temporal Trends in Survival to Adulthood Among Patients Born with Congenital Heart Disease from 1970 to 1992 in Belgium. Circulation.

[89] Patel H., Schaufelberger M., Begley C., Berg M. (2016). Experiences of Health Care in Women with Peripartum Cardiomyopathy in Sweden: A Qualitative Interview Study. BMC Pregnancy Childbirth.

[90] Cooke C.M., Shah A., Kirschenman R.D., Quon A.L., Morton J.S., Care A.S., Davidge S.T. (2018). Increased Susceptibility to Cardiovascular Disease in Offspring Born from Dams of Advanced Maternal Age. J. Physiol..

[91] Rosengren A., Hawken S., Ôunpuu S., Sliwa K., Zubaid M., Almahmeed W.A., Blackett K.N., Sitthi-amorn C., Sato H., Yusuf S. (2004). Association of Psychosocial Risk Factors with Risk of Acute Myocardial Infarction in 11 119 Cases and 13 648 Controls from 52 Countries (the INTERHEART Study): Case-Control Study. Lancet.

[92] American College of Obstetricians and Gynecologists (2023). Screening and Diagnosis of Mental Health Conditions During Pregnancy and Postpartum: ACOG Clinical Practice Guideline No. 4. Obstet. Gynecol..

[93] Regitz-Zagrosek V., Seeland U., Geibel-Zehender A., Gohlke-Bärwolf C., Kruck I., Schaefer C. (2011). Cardiovascular Diseases in Pregnancy. Dtsch. Ärzteblatt Int..

