# Supplementary material for: Impact of Stress and Anxiety on Cardiovascular Health in Pregnancy: A Scoping Review
Source: J Clin Med. 2025 Jan 30;14(3):909. doi: 10.3390/jcm14030909 (PMC11818593; doi:10.3390/jcm14030909)
Supplement: Supplementary file 1 [file jcm-14-00909-s001.zip › jcm-3398435-supplementary.pdf]

Table S1: Quality assessment of cohort and case-control studies using Newcastle-Ottawa Scale.

Study	Selection	Comparability	Outcome	Total
Bilbul et al.	***	**	***	8
Boyer et al.	***	**	**	7
Garza-Volez et al.	***	**	***	8
Horsley et al.	***	**	**	7
Kordi et al.	***	**	**	7
Lackner et al.	***	**	***	8
Lanssens et al.	***	**	**	7
Monk et al.	***	**	***	8
Nath et al.	***	**	***	8
Parisi et al.	***	**	**	7
Thombre et al.	***	**	***	8

Each asterisk is equivalent to one point. The maximum score is 9 (**** for selection, ** for comparability, *** for outcome). Score of 5 to 6 considered as moderate quality and 7 to 9 as high quality.

Table S2. Quality assessment of the cross-sectional studies evaluated by AHRQ

STUDY	Rater	Q1	Q2	Q3	Q4	Q5	Q6	Q7	Q8	Q9	Q10	Q11	TOTAL
Ardani et al.	R1	+	+	+	?	-	+	+	+	NA	-	+	7
	R2	+	+	+	?	+	+	+	+	NA	-	+	8
	C	+	+	+	?	+	+	+	+	NA	-	+	8
Oni et al.	R1	+	+	+	+	-	+	+	+	NA	-	+	8
	R2	+	+	+	?	-	+	+	+	NA	-	+	7
	C	+	+	+	+	-	+	+	+	NA	-	+	8

AHRQ: Agency for Healthcare Research and Quality, Q: Question, R1: Rater 1, R2: Rater 2, C: consensus score, +: Yes, ?: Unclear, -: No, NA: Not applicable.
